# Ileosigmoid knotting: a rare case of intestinal obstruction with intra-operative finding of type II B ileosigmoid knotting

**DOI:** 10.1093/jscr/rjag716

**Published:** 2026-09-01

**Authors:** Fatimah Y Alghareeb, Abdulrahman H Albakheet

**Affiliations:** Department of General Surgery, King Fahd Hospital Hofuf, Prince Salman Road, Al Hufayrah District, Al Hofuf, Al Ahsa, 31982, Saudi Arabia; Department of General Surgery, King Fahd Hospital Hofuf, Prince Salman Road, Al Hufayrah District, Al Hofuf, Al Ahsa, 31982, Saudi Arabia

**Keywords:** ileosigmoid knotting, ileum, sigmoid colon, obstruction

## Abstract

Ileosigmoid knotting is one of the rarest emergencies, occurring when the sigmoid colon or ileum wraps around the base of the other, obstructing the flow. It is considered a rare cause of intestinal obstruction. Clinical signs and symptoms are nonspecific, making preoperative diagnosis challenging. Delays in diagnosis and management may lead to bowel necrosis or peritonitis. Here, we present the case of a 37-year-old male who presented with severe abdominal pain, initially misdiagnosed as adhesive bowel obstruction. ISK was diagnosed intra-operatively.

## Introduction

One of the rarest emergency conditions that results in intestinal closed-loop obstruction is ileosigmoid knotting (ISK), also known as compound volvulus or double volvulus [1]. Both the ileum and the sigmoid colon may be affected. In most cases, the ileum twists around the sigmoid colon, increasing the risk of infection, gangrene, and death [1]. ISK is challenging to diagnose due to its rarity, nonspecific symptoms, radiological findings, and resemblance to sigmoid volvulus [2]. It is typically diagnosed intra-operatively. Early diagnosis and management are essential for better outcomes. Here, we present a case of ISK diagnosed intra-operatively after a misdiagnosis of adhesive bowel obstruction.

## Case report

A 37-year-old male with a history of sleeve gastrectomy three years prior and abdominoplasty one year prior presented to the emergency department with a 2-day history of excruciating lower abdominal pain, nausea, and vomiting. The pain had been intermittently chronic for several months but had acutely progressed. Bowel habits were unchanged. Upon examination, he was vitally stable and oriented, with a soft abdomen exhibiting lower abdominal tenderness. Laboratory workup showed: WBC 12 ×10^9^/l, hemoglobin 16 g/dl, potassium 4 mmol/l, and sodium 141 mmol/l.

**Figure 1:**
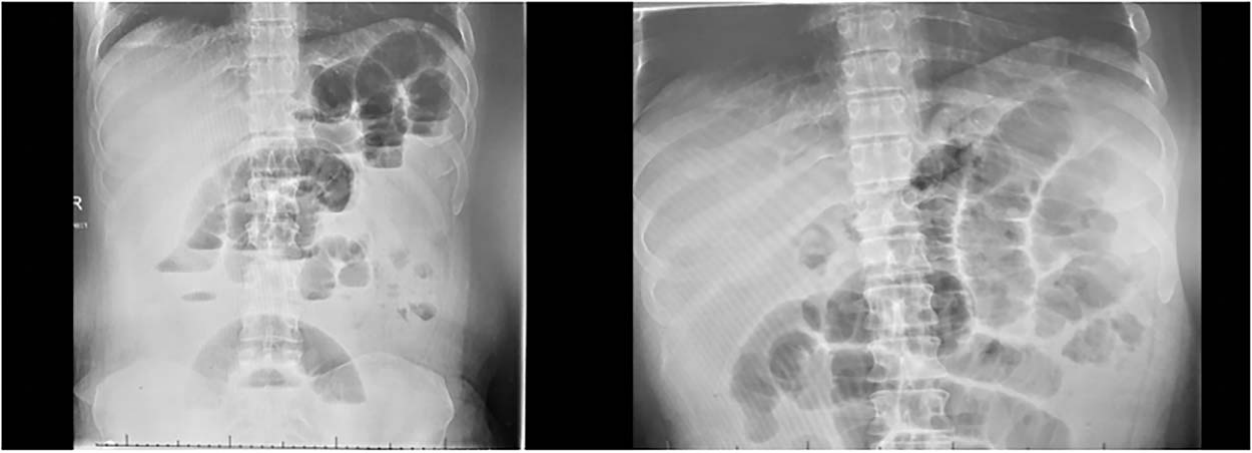
The abdominal X-ray showed an air–fluid level with a dilated small bowel.

**Figure 2:**
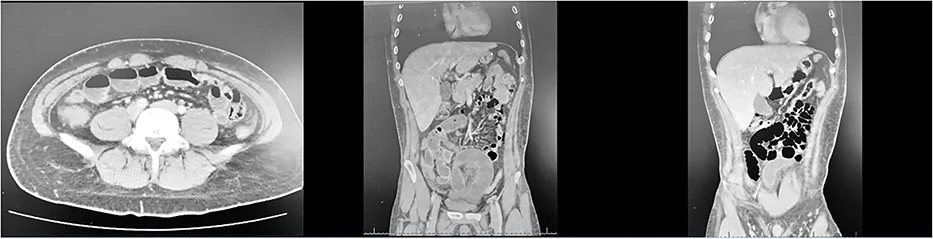
CT abdomen with IV contrast showed a dilated small bowel loop with an air–fluid level of 3.6 cm, a transitional zone in the distal ileum, and a collapsed large bowel loop, without any signs of bowel ischemia.

Abdominal X-ray revealed dilated small bowel loops and air–fluid levels (Fig. 1). Contrast-enhanced computed tomography (CT) of the abdomen and pelvis demonstrated a dilated small bowel loop with a 3.6 cm air–fluid level, a transitional zone in the distal ileum, and a collapsed large bowel without signs of ischemia, suggestive of adhesive bowel obstruction (Fig. 2).

Conservative management via nasogastric tube decompression and intravenous fluids was initiated. However, due to persistent severe pain, difficult defecation, and lack of radiographic improvement, urgent surgical intervention was indicated. Laparoscopic exploration revealed ISK, proximal bowel dilatation, and surrounding adhesions. Due to failure of laparoscopic reduction, the procedure was converted to an open laparotomy via a midline incision.

**Figure 3:**
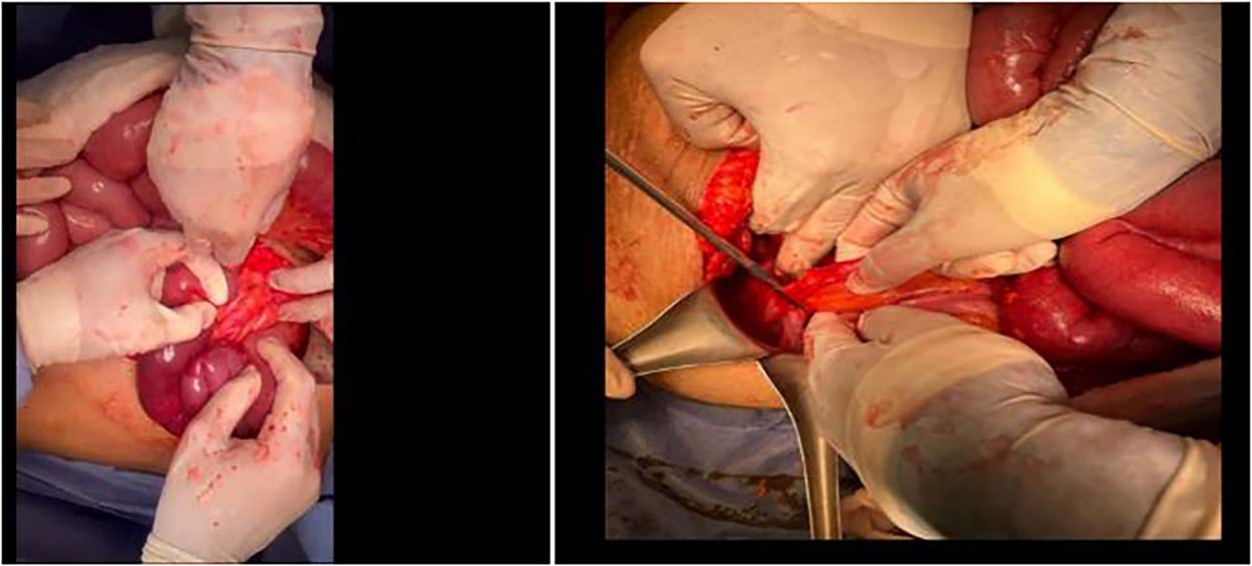
Intra-operative findings included a dilated bowel, internal hernia with a congested bowel that had herniated, ISK, and the sigmoid colon wrapping around the ileum, forming a type II B band.

**Figure 4:**
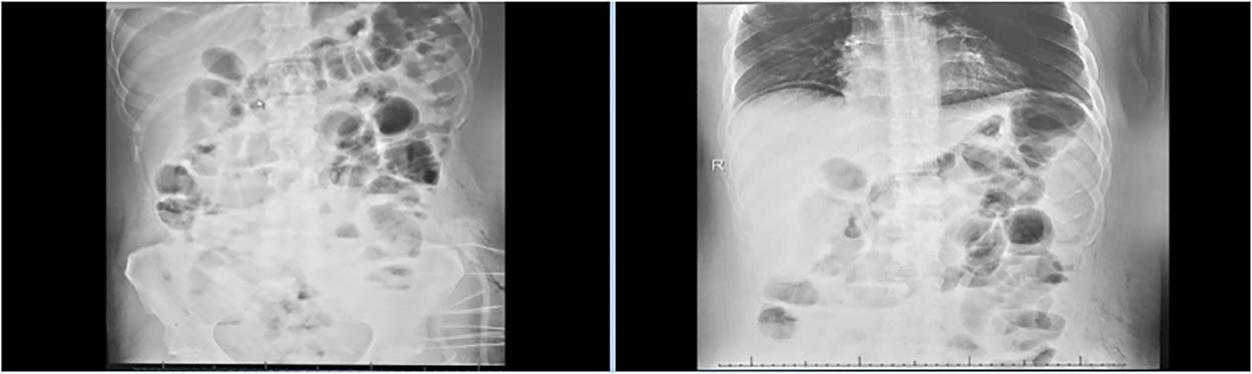
Post-operative abdominal X-ray showed improvement in the dilation.

Adhesiolysis and bowel running from the duodenojejunal junction to the ileocecal valve were performed. An internal hernia with herniation of a congested bowel loop was identified alongside Type IIB ISK, where the sigmoid colon wrapped around the ileum (Fig. 3). The hernia was reduced; following warming, the bowel regained normal perfusion. A small serosal tear was repaired, and the mesenteric defect was closed using 2–0 silk sutures. A drain was placed. Postoperatively, the patient recovered uneventfully, passing flatus and stool, with radiographic improvement shown on follow-up X-ray (Fig. 4). He was discharged on postoperative day three after tolerating oral intake and continued follow-up at the outpatient clinic.

## Discussion

ISK, or double volvulus, is a rare, life-threatening surgical emergency wherein the ileum and sigmoid colon form a knot, causing a closed-loop obstruction that can rapidly progress to gangrene of both segments [1, 3]. First described by Parker in 1845 and later named by Shepherd in 1967 [1], the exact primary etiology of ISK remains unclear [1]. However, several risk factors are well-established. It demonstrates a higher geographic prevalence in regions with a high incidence of sigmoid volvulus, including Asia, Africa, and the Middle East [2, 3]. ISK occurs more frequently in males, typically presenting in the third to fifth decades of life or during the third trimester of pregnancy [4, 5]. Anatomical predispositions include a redundant sigmoid colon with a narrow mesenteric pedicle, a long small bowel mesentery, and a freely mobile ileum [6, 7]. Dietary habits such as high-fiber intake, bulky meals, or a rapid transition from fasting to eating (e.g. during Ramadan) also elevate risk. Additionally, conditions like Meckel’s diverticulitis with thick bands, intussusception, postoperative adhesions, transmesenteric herniation, and malrotation are associated with ISK [1, 2, 6].

Our patient was a male in his third decade with a history of sleeve gastrectomy. This surgical background initially proved misleading, as his presentation was misattributed to adhesive bowel obstruction. Clinically, ISK presents non-specifically with acute abdominal pain, nausea, vomiting, and obstipation, occasionally progressing to shock, sepsis, and diffuse abdominal distension [8, 9].

ISK is categorized into three main anatomical types: Type I involves the ileum wrapping around the sigmoid colon (53.9%–57.5%); Type II occurs when the sigmoid colon wraps around the ileum (18.9%–20.6%); and Type III involves the ileocecal segment wrapping around the sigmoid colon (1.5%) [6, 7, 10–12]. Types I and II are further subdivided into Type A (clockwise, 60%) and Type B (counter-clockwise, 40%) [6, 7, 9]. In our patient, we identified a Type IIB configuration. Furthermore, Atamanalp *et al*. [13] introduced a clinical classification based on preoperative status, risk factors, and surgical findings (Table 1); our patient was classified as C1 (medically free, stable, with viable ileum and sigmoid colon).

**Table 1:** Classification of ISK

C1	C2a	C2b	C3a	C3b	C4a	C4b	C5	C6
A0 D0	One of A, D1. Either older 60 years or present of associated diseases.	Two of A, D1. Older 60 years and present of associated disease.	At most1 of A, D1. Either older 60 years or present of associated diseases.	Two of A, D1. Older 60 years and present of associated disease.	At most1 of A, D1. Either older 60 years or present of associated diseases.	Two of A, D1. Older 60 years and present of associated disease.	–	–
S0	S0 Shock absent	S0 Shock absent	S1 Shock present	S1 Shock present	S0 Shock absent	S0 Shock absent	S1 Shock present	–
G0	G0 Bowel gangrene absent.	G0 Bowel gangrene absent.	G0 Bowel gangrene absent.	G0 Bowel gangrene absent.	G1 Bowel gangrene in ileum or sigmoid.	G1 Bowel gangrene in ileum or sigmoid.	G1 Bowel gangrene in ileum or sigmoid.	G2 Bowel gangrene in ileum and sigmoid.

Adapted from Atamanalp *et al*. [13].

Note: C: class, A (age): A0: under 60 years, A1: 60 years and older. D (associated disease): D0: absent, D1: present. S (shock): S0: absent, S1: present. G (bowel gangrene): G0: absent, G1: present in the ileum or sigmoid colon, G2: in both segments.

Due to non-specific clinical and radiographic features, ISK is usually diagnosed intraoperatively [2]. Plain abdominal X-rays may exhibit multiple air–fluid levels and dilated bowel loops, while CT scans typically demonstrate twisted loops with a mesenteric “whirl sign” [12, 14, 15]. Contrast enemas are contraindicated if perforation is suspected, and endoscopic reduction is strictly avoided due to injury risks [2, 6].

Prompt management involves aggressive fluid resuscitation, electrolyte correction, empirical antibiotics, and nasogastric decompression [8]. Unstable patients may require damage-control surgery with a second-look laparotomy [8], whereas stable patients receive definitive treatment based on bowel viability [2]. Statistically, the small bowel is gangrenous in 75% of cases, and both segments are necrotic in 53%–60%; synchronous viability occurs in only 20%–25% of surgeries [5]. If viable, the knot can be safely untwisted [4], as performed in our case without resection. However, some surgeons perform prophylactic sigmoid colectomy to prevent recurrence [8]. When gangrene is present, untwisting is avoided; primary anastomosis or Hartmann’s procedure is chosen based on the stability of the patient, the distance from the ileocecal valve, and fecal contamination risks [1, 8, 11].

Mortality rates range from 0% to 48%, dictated by sepsis and bowel viability [2, 12]. Common complications include wound dehiscence, infection, and anastomotic leaks [1, 2]. In conclusion, ISK requires a high index of clinical suspicion to ensure early surgical intervention and minimize morbidity and mortality.
